# Vitamin D Reduces Thyroid Cancer Cells Migration Independently From the Modulation of CCL2 and CXCL8 Chemokines Secretion

**DOI:** 10.3389/fendo.2022.876397

**Published:** 2022-04-13

**Authors:** Francesca Coperchini, Alessia Greco, Laura Croce, Elena Petrosino, Beatrice Grillini, Flavia Magri, Luca Chiovato, Mario Rotondi

**Affiliations:** ^1^ Istituti Clinici Scientifici Maugeri IRCCS, Unit of Internal Medicine and Endocrinology, Laboratory for Endocrine Disruptors, Pavia, Italy; ^2^ Department of Internal Medicine and Therapeutics, PHD Course in Experimental Medicine, University of Pavia, Pavia, Italy; ^3^ Department of Internal Medicine and Therapeutics, University of Pavia, Pavia, Italy

**Keywords:** thyroid, tumor microenvironment, chemokines, migration, CCL2 (MCP-1)

## Abstract

**Background:**

Vitamin D3 is largely involved in the regulation of calcium homeostasis. More recently, it was demonstrated that vitamin D exerts several beneficial effects against cancer progression through several mechanisms, including the reduction of cancer cells proliferation and migration. CXCL8 and CCL2 are two chemokines secreted by thyroid tumor cells. In the thyroid tumor microenvironment, these chemokines exert several pro-tumorigenic effects including the one to increase the metastatic potential. The aim of the present study was to investigate if vitamin D could modulate both thyroid cancer cell migration and their ability to secrete CCL2 and CXCL8.

**Methods:**

TPC-1 (RET/PTC rearranged) and 8505C (BRAFV600e mutated) thyroid cancer cell lines were treated with increasing concentrations of 1,25-OH-vitamin D3 (0–1,000 nM). Cell viability was assessed by WST-1 assay, cell migration was evaluated by transwell–migration chamber system, and CCL2 and CXCL8 levels were measured in the cell culture supernatants by ELISA.

**Results:**

Vitamin D did not affect cell viability but reduced, in a dose-dependent and significant manner, thyroid cancer cell migration (ANOVAs *p* < 0.05 for both TPC-1 and 8505C). Vitamin D differently modulated the secretion of CCL2 and CXCL8, by significantly inhibiting the secretion of CCL2 in both thyroid cancer cell lines and inhibiting the secretion of CXCL8 only in TPC-1 (ANOVAs *p* < 0.05).

**Conclusions:**

Vitamin D treatment of thyroid cancer cell lines reduces cell migration independently from the inhibition of the secretion of pro-tumorigenic chemokines. Future studies specifically designed at clarifying the pathways involved in the different inhibitory effects of vitamin D on CCL2 and CXCL8 in thyroid cancer cells appear worthwhile.

## Introduction

Vitamin D is a fat-soluble molecule with a well-established role in skeletal maintenance and calcium metabolism (1).

In recent years, it was shown that vitamin D incubation exerted anti-proliferative, antiangiogenetic, anti-metastatic, pro-differentiation, and pro-apoptotic properties on different types of cancer cells, suggesting its potential beneficial effect against cancer (2–7). Further support for this suggestion was the finding that high serum vitamin D levels were associated with lower cancer incidence (8). On the other hand, vitamin D deficiency may also contribute to the pathogenesis of a number of tumors, being its lower serum concentrations associated with the presence of various types of cancer [colorectal (9), lung (10), and breast (11)] including the thyroid ones (12, 13). Making a focus on thyroid cancer, several preclinical studies have demonstrated a beneficial effect of vitamin D administration also on this type of tumor, indeed: i) *in vitro*, the active form of vitamin D (1,25(OH)_2_D3) and one of its analogs (MART-10) inhibited thyroid cancer cell migration and invasion (14); ii) vitamin D treatment reduced the proliferation of thyroid cancer stem cells (15); iii) in SCID mice transfected with human thyroid follicular carcinoma-derived cells, supplementation of calcitriol reduced tumor size, increased cellular differentiation, and prevented metastasization (16); vi) in the same mouse model, treatment with 1,25(OH)_2_D and its non-calcimimetic analog EB1089 resulted in a 50% reduction in tumor weight (16). Vitamin D counteracts tumor progression interacting with not only normal and cancer cells but also cancer-associated stromal cells and cancer stem cells within the tumor microenvironment by upregulating (tumor suppressors genes, TGFβ, and PDF) or downregulating (STAT3, NF-kB, TNFα, cyclins, and oncogenes) different factors that result in anti-inflammatory, anti-angiogenic, anti-proliferative, pro-apoptotic, and pro-differentiating events (17, 18).

Chemokines are soluble mediators largely secreted within the tumor microenvironment, which crucially contribute to a more or less aggressive clinical behavior of cancer (19–21). CXCL8 and CCL2 are two chemokines playing important roles in driving the progression of different types of cancer. CXCL8 is the most studied pro-tumorigenic chemokine promoting cancer progression, angiogenesis, and metastasis, whose role was recently deeply investigated also in thyroid cancer (21–24). CCL2 was discovered to be produced by several tumor cells including thyroid cancer cells, and hence its known functions include regulation of tumor angiogenesis, metastasization, and immune response (24–28).

The ability of vitamin D to reduce CXCL8 expression was demonstrated in prostate cancer cells (28), with the consequence of a reduction of angiogenesis, one of the CXCL8 well-known pro-tumorigenic effects. No data are available about vitamin D potential effects on CCL2 secretion. The hypothesis that vitamin D could affect the CXCL8 and/or CCL2 secretion was never tested in thyroid cancer.

The aim of the present study was to investigate whether vitamin D (1,25-OH_2_ D3) exerts both direct (cell viability and migration) and indirect (modulation of the pro-tumorigenic chemokines and CXCL8 and/or CCL2 secretion) antitumor effects in TPC-1 and 8505C thyroid cancer cell lines.

## Materials and Methods

### Thyroid Tumor Cell Lines 8505C and TPC-1

Human thyroid cancer cell lines, 8505C harboring the BRAFV600E mutation and TPC-1 bearing the RET/PTC rearrangement, were used for these experiments. These cell lines had been previously tested and authenticated by DNA analysis. Cancer cells were propagated in Dulbecco’s Modified Eagle Medium (DMEM) and Roswell Park Memorial Institute (RPMI) (Sigma, Saint Louis, MO, USA) supplemented with 10% fetal bovine serum (Sigma, Saint Louis, MO, USA), 2 mM of l-glutamine, and 100 U/ml of penicillin/streptomycin (Sigma, Saint Louis, MO, USA). Cells were incubated with the chosen stimuli in a serum-free medium.

### Cell Viability and WST-1 Assay

8505C and TPC-1 were grown in a complete medium until an 80% confluence was reached. Cells were then detached and seeded in 96-well flat plates at a density of 2 × 10^4^ cells/well. Complete medium was supplemented with increasing concentrations of vitamin D (1,25-OH_2_ D3) (0, 0.1, 1, 10, 100, and 1,000 nM). The incubation time was 24 h. At the end of treatment, 20 µl of WST-1 was added to the wells; plates were then incubated for 30 min at 37°C in a 5% CO_2_ atmosphere. WST-1 is a colorimetric reagent, which, after cleavage of a tetrazolium salt, MTS, by mitochondrial dehydrogenases, results in the production of formazan by viable cells only. Absorbance was then measured at 450 nm by using a multimode plate reader (Victor NIVO nt, PerkinElmer, Waltham, MA, USA).

### Migration

The cell migration assay was performed with the transwell migration chamber system (Merck Millipore, Milan, Italy). Briefly, 8505C and TPC-1 were cultured for 24 h with fresh medium alone or supplemented with vitamin D (1,25-OH_2_ D3) 1,000 nM. After treatment, 20 × 10^3^ cells/well were seeded in the upper chambers of 96-well plates with polycarbonate inserts having 0.3 cm^2^/well membrane area and 8-μm pore size. In each condition, the lower chambers were filled with 150 μl of the corresponding medium. Cells were left to migrate for 16 h at 37°C and 5% CO_2_. At the end of the incubation, samples were analyzed as previously described (29). Briefly, cell inserts were placed in May-Grünwald Stain for 3 min and then placed in May-Grünwald Stain diluted 1:5 for the other 3 min. Cell inserts were subsequently washed in distilled water and placed in Giemsa solution for 5 min. Finally, cell inserts were washed again with distilled water. Three replicates were evaluated for each condition. Images were acquired using an Olympus BX51 microscope (Olympus, Deutschland GmbH, Hamburg, Germany). The number of migrated cells was counted by analyzing 12 random fields of the membranes per condition. Data are expressed as % of mean numbers of migrated cells ± SD.

### Wound Healing Assay

For wound healing assay cells were seeded in a 6-well plate. When cells reached nearly 90% of cell confluence, cells were incubated in a serum-free medium overnight to avoid wound closure due to cell proliferation. Then, a scratch wound was created in the monolayer using a sterile 200-µl pipette tip. Cells were then treated with fresh medium alone or supplemented with 1,000 nM of vitamin D (1,25-OH_2_ D3). Phase-contrast images were captured between 0 and 24 h using an Olympus IX53 microscope (Olympus, Deutschland GmbH, Hamburg, Germany). Data are expressed as the percentages of the remaining gap area after 24 h relative to the initial gap area (0 h). The area was measured using the LCmicro software (Olympus Soft Imaging Solutions GmbH).

### Modulation of CXCL8 Secretion in 8505C and TPC-1 Cell Lines in the Presence or Absence of Increasing Concentrations of Vitamin D

For the CXCL8 secretion assays, 3,000 cells were seeded into 96-well plates in a complete medium. After adherence to the plastic surface, 8505C and TPC-1 cells were incubated for 24 h in a serum-free medium (basal condition) with increasing concentrations of vitamin D (1,25-OH_2_ D3) (0, 0.1, 1, 10, 100, and 1,000 nM) (Sigma, Saint Louis, MO, USA). All experiments were performed in triplicates.

### Modulation of CCL2 Secretion in 8505C and TPC-1 Cell Lines in the Presence or Absence of Increasing Concentrations of Vitamin D

For the CCL2 secretion assays, 30,000 cells were seeded into 96-well plates in a complete medium. After adherence to the plastic surface, 8505C and TPC-1 cells were incubated for 24 h in a serum-free medium (basal condition) with increasing concentrations of vitamin D (1,25-OH_2_ D3) (0, 0.1, 1, 10, 100, and 1,000 nM) (Sigma, Saint Louis, MO, USA). All experiments were performed in triplicates.

### ELISA for CXCL8 and CCL2

CXCL8 was measured in cell supernatants of 8505C and TPC-1 using commercially available kits (R&D Systems, Minneapolis, MN, USA). The mean minimum detectable concentration of CXCL8 was 3.5 pg/ml. The intra- and inter-assay coefficients of variation were 3.4% and 6.8%, respectively. Samples were assayed in duplicates. CCL2 was measured in cell supernatants of TPC-1 and 8505C using commercially available kits (R&D Systems, Minneapolis, MN, USA). The mean minimum detectable concentration of CCL2 was 31.2 pg/ml. The intra- and inter-assay coefficients of variation were 4.7% and 5.8%, respectively. Samples were assayed in duplicates.

### Statistical Analysis

Statistical analysis was performed using the SPSS software (SPSS, Inc., Evanston, IL, USA). Mean group values were compared by using one-way ANOVA for normally distributed variables. *Post-hoc* analysis was performed according to Bonferroni’s correction for multiple comparisons. Between-group comparisons were performed by means of Student’s t-test for unpaired data. Values are reported as mean ± SD unless otherwise noted. A *p*-value <0.05 was considered statistically significant.

## Results

### Vitamin D Effect on Thyroid Cancer Cell Viability

**Figure 1:**
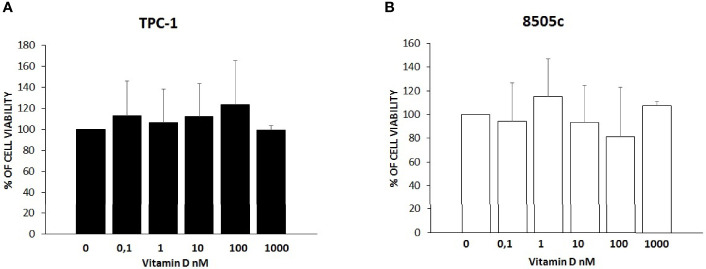
Effects of vitamin D on thyroid cancer cell viability. **(A)** Treatment with vitamin D at increasing concentrations did not affect cell viability of TPC-1 (ANOVA F = 0.417, *p* = 0.831). **(B)** Treatment with vitamin D at increasing concentrations did not affect cell viability of 8505C (ANOVA F = 1.067, *p* = 0.411) **(B)**.

To rule out a potential cytotoxic effect of vitamin D, a cell viability assay was performed for both TPC-1 and 8505C. As shown in **Figure 1**, treatment with vitamin D at increasing concentrations did not affect cell viability of TPC-1 (ANOVA F = 0.417, *p* = 0.831) (**Figure 1A**) and 8505C (ANOVA F = 1.067, *p* = 0.411) (**Figure 1B**).

### Vitamin D Reduces Thyroid Cancer Cell Migration

**Figure 2:**
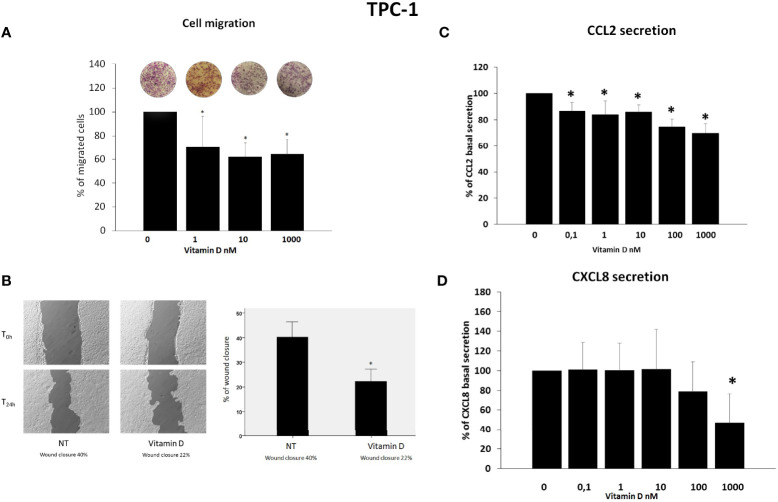
Effects of vitamin D on TPC-1 thyroid cancer cell migration and on the secretion of CCL2 and CXCL8. **(A)** Vitamin D reduced the basal TPC-1 cell migration (ANOVA F = 27.664; *p* < 0.001). **(B)** Representative images and histogram of three independent experiments of wound healing assay: after 24 h (T24h), TPC-1 cell migration produced a wound closure of 40% (NT = not treated cells) as compared to the scratch time (T0), and the treatment of vitamin D (1,000 nM) produced less closure of the wound after 24 h (T24h) (vitamin D 22%) as compared to the scratch time (T0). Data are expressed as the percentages of the remaining gap area after 24 h relative to the initial gap area (0 h). **(C)** The treatment with vitamin D inhibited the CCL2 secretion in TPC-1 starting from a concentration of 0.1 nM of vitamin D (ANOVA F = 15.303; *p* < 0.0001). **(D)** Vitamin D inhibited the secretion of CXCL8 only at the maximal concentration of 1,000 nM of vitamin D (ANOVA F = 7.4; *p* < 0.0001). *p < 0.05 vs. untreated.

**Figure 3:**
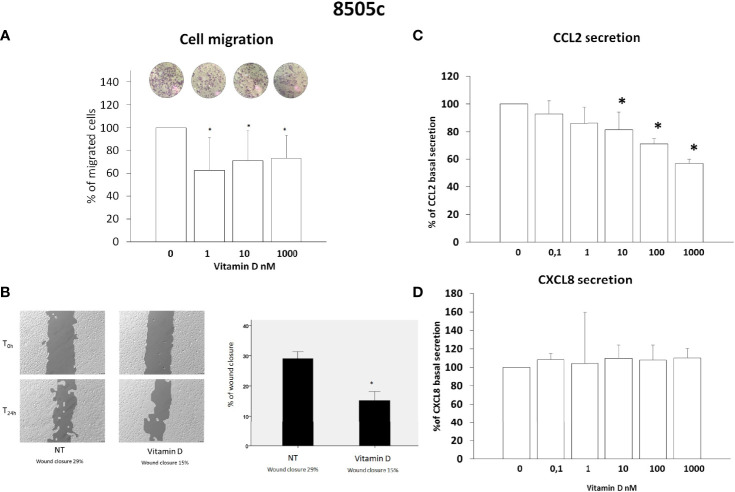
Effects of vitamin D on 8505C thyroid cancer cell migration and on the secretion of CCL2 and CXCL8. **(A)** Vitamin D reduced the spontaneous 8505C cell migration (ANOVA F = 6.268; *p* < 0.001). **(B)** Representative images and histogram of three independent experiments of wound healing assay: after 24 h (T24h), 8505C cell migration produced a wound closure of 29% (NT not treated cells) as compared to the scratch time (T0), the treatment of vitamin D (1,000 nM) produced less closure of the wound after 24 h (T24h) (vitamin D 15%) as compared to the scratch time (T0). Data are expressed as the percentages of the remaining gap area after 24 h relative to the initial gap area (0 h). **(C)** The treatment with vitamin D inhibited the CCL2 secretion in 8505C starting from a concentration of 10 nM of vitamin D (F = 20.9103; *p* < 0.0001). **(D)** Vitamin D did not inhibit the CXCL8 secretion (ANOVA F = 1.1; NS). *p < 0.05 vs. untreated.

Results of migration assay showed that vitamin D reduced the spontaneous TPC-1 cell migration (ANOVA F = 27.664; *p* < 0.001) (**Figure 2A**) and also the spontaneous 8505C cell migration (ANOVA F = 6.268; *p* < 0.001) (**Figure 3A**). Results are expressed as % of migrated cells calculated according to the untreated samples estimated as 100%. Results were confirmed by wound healing assay. Vitamin D produced a significant reduction of wound closure after 24 h in TPC-1 cell (Students’ t-test; *p* < 0.05) (**Figure 2B**) and also in 8505C cell (Students’ t-test; *p* < 0.05) (**Figure 3B**).

### Vitamin D Inhibits CCL2 and CXCL8 Secretion in TPC-1 Cancer Cells

CCL2 and CXCL8 concentrations were assayed in the supernatants of TPC-1 thyroid cancer cell lines. In TPC-1 cells, the treatment with vitamin D inhibited the CCL2 secretion (ANOVA F = 15.303; *p* < 0.0001) (**Figure 2C**). *Post-hoc* analysis by Bonferroni evidenced a significant inhibition of the CCL2 secretion starting from a concentration of 0.1 nM of vitamin D (*p* < 0.05 vs. basal). Vitamin D inhibited also the secretion of CXCL8 even if a significant inhibition was observed only at the maximal concentration of 1,000 nM of vitamin D (**Figure 2D**) (ANOVA F = 7.4; *p* < 0.0001; *Post-hoc p* < 0.001 vs. basal).

### Vitamin D Inhibits CCL2 But Not CXCL8 Secretion in 8505C Cancer Cells

CCL2 and CXCL8 concentrations were assayed in the supernatants of 8505C thyroid cancer cell lines. In 8505C cells, the treatment with vitamin D inhibited the CCL2 secretion ANOVA (F = 20.9103; *p* < 0.0001); in contrast with TPC-1 cells, the inhibition started from concentration above 10 nM of vitamin D (**Figure 3C**). However, in 8505C cells, the treatment with vitamin D did not inhibit the CXCL8 secretion (ANOVA F = 1.1; NS) at any of the tested concentrations (**Figure 3D**).

## Discussion

The results of the present *in vitro* study showed that treatment with vitamin D is able to reduce thyroid cancer cell migration in a dose-dependent and significant manner. Furthermore, vitamin D differently modulates the secretion of CCL2 and CXCL8 by thyroid cancer cell lines. In particular, the secretion of CCL2 was inhibited by vitamin D in both TPC-1 (RET/PTC) and 8505C (BRAFV600e) thyroid cancer cell lines. In contrast, inhibition of the CXCL8 secretion was only observed with high doses of vitamin D and only in TPC-1 (RET/PTC) cells.

Thus, the here reported results allow extending the spectrum of the antitumor effects of vitamin D to its ability to reduce the secretion of pro-tumorigenic chemokines.

There is convincing evidence in the literature that vitamin D is able to affect cancer progression at several levels by affecting tumor microenvironment composition consequently reducing cancer cell growth, inhibiting angiogenesis, and interfering with metastatic processes (30, 31).

Previous *in vitro* and *in vivo* results showed that the reduction of CXCL8 and CCL2 secretion inhibits some of the pro-tumorigenic effects mediated by both these chemokines in thyroid cancer (32). The results of migration assay experiments would confirm the anti-metastatic effects of vitamin D on thyroid cancer cell lines. However, the fact that basal cell migration occurred in a dose-dependent manner and was observed also for concentrations of vitamin D at which no inhibitory effect on chemokine secretion was demonstrated would support that the anti-migration effect of vitamin D would depend upon other mechanisms rather than by its ability to inhibit the secretion of CCL2 and CXCL8, chemokines that are known to be crucially involved in the metastatic process (27, 32–34).

In particular, the results indicate a more consistent inhibitory effect of vitamin D on CCL2 secretion, which was observed in both thyroid cell types and at several concentrations tested, as compared to CXCL8, which was reduced only in TPC-1 and only by using the highest concentration of vitamin D. This observation would indicate that different pathways are involved in the secretion of CXCL8 and CCL2 in thyroid cancer cells.

The ability of vitamin D to reduce the secretion of CCL2 in TPC-1 and 8505C cells should not be underestimated, in view of the several pro-tumorigenic effects exerted by CCL2 within the tumor microenvironment and by the therapeutic benefits produced by strategies aimed at reducing its biological effect. Briefly, it was demonstrated that i) CCL2 attracts macrophages in the tumor microenvironment, which ultimately favor cancer progression (35); ii) CCL2 is involved in the metastatic process in several cancers, being its neutralization with anti-CCL2 antibodies associated with a reduction of the metastatic ability (36); and iii) the blockade of CCL2/CCR2 axis produces therapeutic effects in a mouse model of hepatocellular carcinoma (37).

The dose–response curves obtained for testing the efficacy of increasing concentrations of vitamin D in reducing CCL2 secretion would indicate that the inhibitory effect is higher in TPC-1 as compared to 8505C cells. The finding that BRAF mutated vs. RET/PTC rearranged thyroid cells would differently respond to vitamin D treatment is of potential interest and deserves to be discussed.

Indeed, previous *in vitro* studies demonstrated that the BRAFV600E mutation is associated with a more severe disruption of normal vitamin D signaling as compared to cancer cells harboring other genetic abnormalities such as RET/PTC (38).

The fact that vitamin D might be more or less effective in producing anticancer effects according to the specific type of cancer cell line was previously reported also for its ability to reduce thyroid cancer cell growth (4, 18, 19). In particular, Zou et al. reported that there are some cell lines (like TPC-1) that are more sensitive to vitamin D as compared with others (like 8505C) that are more resistant to vitamin D treatment (38).

The results of the present study would rule out the possibility that the reduction of cell migration, induced by vitamin D, would stem from its ability to inhibit the secretion of CXCL8 and CCL2. As a result, it is clear that other mechanisms must be involved. The design of the present study does not allow drawing mechanistic conclusions as to this aspect. Nevertheless, according to previous studies addressing the ability of vitamin D in inhibiting cell migration of different cancer cell types, a few considerations could be done. Indeed, it was previously demonstrated that vitamin D is able to limit the epithelial-to-mesenchymal transition (EMT), by both downregulating molecules favoring cell migration and upregulating several inhibitory factors involved in the EMT process (39). Furthermore, studies in breast cancer cells demonstrated that vitamin D promotes the formation of focal adhesions by increasing the expression and phosphorylation of focal adhesion kinase (FAK) (40).

Furthermore, in ovarian cancer cells, vitamin D suppressed EMT through the reduction of Slug and Snail (two transcription factors favoring EMT) and the upregulation of E-cadherin (41).

As far as thyroid cancer is concerned, Chiang et al. demonstrated that vitamin D was able to both upregulate E-cadherin and repress N-cadherin expression, leading to the inhibition of cell migration. In addition, vitamin D also inhibited MMP-9 secretion by extracellular matrix, further reducing cancer cell migration (14).

Taking together the above-summarized evidence, it seems clear that it is likely that more than one mechanism accounts for the inhibitory effect on cancer cell migration exerted by vitamin D.

In conclusion, the results of the present study support both direct and indirect antitumor activities of vitamin D in thyroid cancer cells. Indeed, on the one hand, vitamin D directly reduces thyroid cancer cell migration; on the other hand, vitamin D reduces the secretion of chemokines with a well-established role in driving thyroid cancer progression.

Future studies specifically addressing this issue will be required in order to further characterize the antitumor effects of vitamin D.

## Data Availability Statement

The raw data supporting the conclusions of this article will be made available by the authors, without undue reservation.

## Author Contributions

FC conceptualized the study. FC, LCr, AG, EP, and BG wrote and prepared the original draft. MR, LCh, and FM wrote, reviewed, and edited the paper. MR supervised the study. All authors contributed to the article and approved the submitted version.

## Conflict of Interest

The authors declare that the research was conducted in the absence of any commercial or financial relationships that could be construed as a potential conflict of interest.

## Publisher’s Note

All claims expressed in this article are solely those of the authors and do not necessarily represent those of their affiliated organizations, or those of the publisher, the editors and the reviewers. Any product that may be evaluated in this article, or claim that may be made by its manufacturer, is not guaranteed or endorsed by the publisher.

## References

[1] Lips P. Vitamin D Physiology. Prog Biophys Mol Biol (2006) 92(1):4–8. doi: 10.1016/j.pbiomolbio.2006.02.016 16563471

[2] Deeb KK Trump DL Johnson CS. Vitamin D Signalling Pathways In Cancer: Potential For Anticancer Therapeutics. Nat Rev Cancer (2007) 7(9):684–700. doi: 10.1038/nrc2196 17721433

[3] Chiang KC Yeh CN Chen MF Chen TC. Hepatocellular Carcinoma And Vitamin D: A Review. J Gastroenterol Hepatol (2011) 26(11):1597–603. doi: 10.1111/j.1440-1746.2011.06892.x 21880026

[4] Chiang KC Yeh CN Chen TC. Vitamin D And Pancreatic Cancer-An Update. Cancers (Basel) (2011) 3(1):213–26. doi: 10.3390/cancers3010213 PMC3756357 24212614

[5] Chiang KC Chen TC. The Anti-Cancer Actions Of Vitamin D. Anticancer Agents Med Chem (2013) 13(1):126–39. doi: 10.2174/187152013804487443 23094926

[6] Feldman D Krishnan AV Swami S Giovannucci E Feldman BJ. The Role Of Vitamin D In Reducing Cancer Risk And Progression. Nat Rev Cancer (2014) 14(5):342–57. doi: 10.1038/nrc3691 24705652

[7] Migliaccio S Di Nisio A Magno S Romano F Barrea L Colao AM. Vitamin D Deficiency: A Potential Risk Factor For Cancer In Obesity? Int J Obes (Lond) (2022) 46(4):707–17. doi: 10.1038/s41366-021-01045-4 35027681

[8] Bade B Zdebik A Wagenpfeil S Gräber S Geisel J Vogt T. Low Serum 25-Hydroxyvitamin D Concentrations Are Associated With Increased Risk For Melanoma And Unfavourable Prognosis. PloS One (2014) 9(12):E112863. doi: 10.1371/journal.pone.0112863 25437008 PMC4249825

[9] Song M Nishihara R Wang M Chan AT Qian ZR Inamura K. Plasma 25-Hydroxyvitamin D And Colorectal Cancer Risk According To Tumour Immunity Status. Gut (2016) 65(2):296–304. doi: 10.1136/gutjnl-2014-308852 25591978 PMC4503524

[10] Sharma K Goehe RW Di X Hicks MA Torti SV Torti FM. A Novel Cytostatic Form Of Autophagy In Sensitization Of Non-Small Cell Lung Cancer Cells To Radiation By Vitamin D And The Vitamin D Analog, Eb 1089. Autophagy (2014) 10(12):2346–61. doi: 10.4161/15548627.2014.993283 PMC4502704 25629933

[11] Imtiaz S Siddiqui N. Vitamin-D Status At Breast Cancer Diagnosis: Correlation With Social And Environmental Factors And Dietary Intake. J Ayub Med Coll Abbottabad (2014) 26(2):186–90. 25603674

[12] Kim JR Kim BH Kim SM Oh MY Kim WJ Jeon YK. Low Serum 25 Hydroxyvitamin D Is Associated With Poor Clinicopathologic Characteristics In Female Patients With Papillary Thyroid Cancer. Thyroid (2014) 24(11):1618–24. doi: 10.1089/thy.2014.0090 25133449

[13] Kim D. The Role Of Vitamin D In Thyroid Diseases. Int J Mol Sci (2017) 18(9):1949. doi: 10.3390/ijms18091949 PMC5618598 28895880

[14] Chiang KC Kuo SF Chen CH Ng S Lin SF Yeh CN. Mart-10, The Vitamin D Analog, Is A Potent Drug To Inhibit Anaplastic Thyroid Cancer Cell Metastatic Potential. Cancer Lett (2015) 369(1):76–85. doi: 10.1016/j.canlet.2015.07.024 26282787

[15] Peng W Wang K Zheng R Derwahl M. 1,25 Dihydroxyvitamin D3 Inhibits The Proliferation Of Thyroid Cancer Stem-Like Cells *Via* Cell Cycle Arrest. Endocr Res (2016) 41(2):71–80. doi: 10.3109/07435800.2015.1037048 27030645

[16] Dackiw AP Ezzat S Huang P Liu W Asa SL. Vitamin D3 Administration Induces Nuclear P27 Accumulation, Restores Differentiation, And Reduces Tumor Burden In A Mouse Model Of Metastatic Follicular Thyroid Cancer. Endocrinology (2004) 145(12):5840–6. doi: 10.1210/en.2004-0785 15319350

[17] Díaz L Díaz-Muñoz M García-Gaytán AC Méndez I. Mechanistic Effects Of Calcitriol In Cancer Biology. Nutrients (2015) 7(6):5020–50. doi: 10.3390/nu7065020 PMC4488829 26102214

[18] Wu X Hu W Lu L Zhao Y Zhou Y Xiao Z. Repurposing Vitamin D For Treatment Of Human Malignancies. Acta Pharm Sin B (2019) 9(2):203–19. doi: 10.1016/j.apsb.2018.09.002 PMC6437556 30972274

[19] Cunha LL Marcello MA Ward LS. The Role Of The Inflammatory Microenvironment In Thyroid Carcinogenesis. Endocr Relat Cancer (2014) 21(3):R85–R103. doi: 10.1530/ERC-13-0431 24302667

[20] Balkwill FR. The Chemokine System And Cancer. J Pathol (2012) 226(2):148–57. doi: 10.1002/path.3029 21989643

[21] Rotondi M Coperchini F Latrofa F Chiovato L. Role Of Chemokines In Thyroid Cancermicroenvironment: Is Cxcl8 The Main Player? Front Endocrinol (2018) 9:314. doi: 10.3389/fendo.2018.00314 PMC6021500 29977225

[22] Rotondi M Coperchini F Chiovato L. Cxcl8 In Thyroid Disease: From Basic Notions To Potential Applications In Clinical Practice. Cytokine Growth Factor Rev (2013) 24(6):539–46. doi: 10.1016/j.cytogfr.2013.08.001 24011840

[23] Rotondi M Coperchini F Pignatti P Sideri R Groppelli G Leporati P. Interferon-Γ And Tumor Necrosis Factor-A Sustain Secretion Of Specific Cxc Chemokines In Human Thyrocytes: A First Step Toward A Differentiation Between Autoimmune And Tumor-Related Inflammation? J Clin Endocrinol Metab (2013) 98(1):308–13. doi: 10.1210/jc.2012-2555 23118425

[24] Coperchini F Croce L Marinò M Chiovato L Rotondi M. Role Of Chemokine Receptors In Thyroid Cancer And Immunotherapy. Endocr Relat Cancer (2019) 26(8):R465–78. doi: 10.1530/ERC-19-0163 31146261

[25] Fei L Ren X Yu H Zhan Y. Targeting The Ccl2/Ccr2 Axis In Cancer Immunotherapy: One Stone, Three Birds? Front Immunol (2021) 12:771210. doi: 10.3389/fimmu.2021.771210 34804061 PMC8596464

[26] Xu M Wang Y Xia R Wei Y Wei X. Role Of The Ccl2-Ccr2 Signalling Axis In Cancer: Mechanisms And Therapeutic Targeting. Cell Prolif (2021) 54(10):E13115. doi: 10.1111/cpr.13115 34464477 PMC8488570

[27] Kadomoto S Izumi K Mizokami A. Roles Of Ccl2-Ccr2 Axis In The Tumor Microenvironment. Int J Mol Sci (2021) 22(16):8530. doi: 10.3390/ijms22168530 34445235 PMC8395188

[28] Bao BY Yao J Lee YF. 1alpha, 25-Dihydroxyvitamin D3 Suppresses Interleukin-8-Mediated Prostate Cancer Cell Angiogenesis. Carcinogenesis (2006) 27(9):1883–93. doi: 10.1093/carcin/bgl041 16624828

[29] Abbonante V Gruppi C Rubel D Gross O Moratti R Balduini A. Discoidin Domain Receptor 1 Protein Is A Novel Modulator Of Megakaryocyte-Collagen Interactions. J Biol Chem (2013) 288(23):16738–46. doi: 10.1074/jbc.M112.431528 PMC3675607 23530036

[30] Kang Z Wang C Tong Y Li Y Gao Y Hou S. Novel Nonsecosteroidal Vitamin D Receptor Modulator Combined With Gemcitabine Enhances Pancreatic Cancer Therapy Through Remodeling Of The Tumor Microenvironment. J Med Chem (2021) 64(1):629–43. doi: 10.1021/acs.jmedchem.0c01197 33381963

[31] Fraga M Yáñez M Sherman M Llerena F Hernandez M Nourdin G. Immunomodulation Of T Helper Cells By Tumor Microenvironment In Oral Cancer Is Associated With Ccr8 Expression And Rapid Membrane Vitamin D Signaling Pathway. Front Immunol (2021) 12:643298. doi: 10.3389/fimmu.2021.643298 34025655 PMC8137990

[32] Passaro C Borriello F Vastolo V Di Somma S Scamardella E Gigantino V. The Oncolytic Virus Dl922-947 Reduces Il-8/Cxcl8 And Mcp-1/Ccl2 Expression And Impairs Angiogenesis And Macrophage Infiltration In Anaplastic Thyroid Carcinoma. Oncotarget (2016) 7(2):1500–15. doi: 10.18632/oncotarget.6430 PMC4811476 26625205

[33] Ferrari SM Elia G Piaggi S Baldini E Ulisse S Miccoli M. Ccl2 Is Modulated By Cytokines And Ppar-G In Anaplastic Thyroid Cancer. Anticancer Agents Med Chem (2017) 18(3):458–66. doi: 10.2174/1871520617666170719152349 28730964

[34] Rotondi M Coperchini F Latrofa F Chiovato L. Role Of Chemokines In Thyroid Cancer Microenvironment: Is Cxcl8 The Main Player? Front Endocrinol (Lausanne) (2018) 9:314. doi: 10.3389/fendo.2018.00314 29977225 PMC6021500

[35] Mizutani K Sud S Mcgregor NA Martinovski G Rice BT Craig MJ. The Chemokine Ccl2 Increases Prostate Tumor Growth And Bone Metastasis Through Macrophage And Osteoclast Recruitment. Neoplasia (2009) 11(11):1235–42. doi: 10.1593/neo.09988 PMC2767225 19881959

[36] Qian BZ Li J Zhang H Kitamura T Zhang J Campion LR. Ccl2 Recruits Inflammatory Monocytes To Facilitate Breast-Tumour Metastasis. Nature (2011) 475(7355):222–5. doi: 10.1038/nature10138 PMC3208506 21654748

[37] Teng KY Han J Zhang X Hsu SH He S Wani NA. Blocking The Ccl2-Ccr2 Axis Using Ccl2-Neutralizing Antibody Is An Effective Therapy For Hepatocellular Cancer In A Mouse Model. Mol Cancer Ther (2017) 16(2):312–22. doi: 10.1158/1535-7163.MCT-16-0124 PMC5292068 27980102

[38] Zou M Binhumaid FS Alzahrani AS Baitei EY Al-Mohanna FA Meyer BF. Increased Cyp24a1 Expression Is Associated With Braf(V600e) Mutation And Advanced Stages In Papillary Thyroid Carcinoma. Clin Endocrinol (Oxf) (2014) 81(1):109–16. doi: 10.1111/cen.12396 24382015

[39] Larriba MJ García De Herreros A Muñoz A. Vitamin D And The Epithelial To Mesenchymal Transition. Stem Cells Int (2016) 2016:6213872. doi: 10.1155/2016/6213872 26880977 PMC4736588

[40] Pendás-Franco N González-Sancho JM Suárez Y Aguilera O Steinmeyer A Gamallo C. Vitamin D Regulates The Phenotype Of Human Breast Cancer Cells. Differentiation (2007) 75(3):193–207. doi: 10.1111/j.1432-0436.2006.00131.x 17288543

[41] Hou YF Gao SH Wang P Zhang HM Liu LZ Ye MX. 1α,25(Oh)_2_D_3_ Suppresses The Migration Of Ovarian Cancer Skov-3 Cells Through The Inhibition Of Epithelial-Mesenchymal Transition. Int J Mol Sci (2016) 17(8):1285. doi: 10.3390/ijms17081285 PMC5000682 27548154

